# Modeling menstrual cycle phases from urinary hormone trajectories using an autoregressive hidden Markov model

**DOI:** 10.1371/journal.pdig.0001502

**Published:** 2026-11-05

**Authors:** Theresa M. Stujenske, Thomas P. Bouchard, Alexis Troy, Sara Kelemen, Brianna Folino, Tara Wills, Lauren A. Sugden

**Affiliations:** 1 School of Nursing, Duquesne University, Pittsburgh, Pennsylvania, United States of America; 2 Center for Fertility Awareness Education and Research, Nasuti College of Osteopathic Medicine, Duquesne University, Pennsylvania, United States of America; 3 Department of Family Medicine, University of Calgary, Calgary, Canada; 4 Department of Mathematics and Computer Science, Duquesne University, Pittsburgh, Pennsylvania, United States of America; Xinjiang Medical University Affiliated First Hospital, CHINA

## Abstract

The recent availability of at-home menstrual cycle tracking technology has created opportunities for personalized assessment of reproductive health, alongside improved characterization of hormone patterns in women with and without reproductive disorders such as polyendocrine metabolic ovarian syndrome (PMOS), which affects approximately 10% of reproductive-age women. In this study, we leverage self-tracked urinary hormone data to develop an autoregressive Hidden Markov model (arHMM) that maps cycle days to physiologically meaningful phases based on hormone trajectories. By modeling day-to-day hormonal dynamics rather than absolute hormone levels, and allowing variable phase durations, this approach accommodates substantial variability in menstrual cycles, thereby enabling meaningful comparisons within and between individuals. Across more than 3800 cycles from over 1100 individuals, we find that arHMM-derived phases reproduce expected hormonal patterns within follicular, periovulatory, and luteal phases, and that phase-based timing for hormone testing outperforms conventional cycle day–based testing in capturing the luteinizing hormone surge and post-ovulatory progesterone rise, highlighting limitations of fixed-day clinical protocols. We identify phase-specific differences between healthy controls and individuals with self-reported PMOS, including lower luteinizing hormone in the periovulatory phase, and reduced luteal phase progesterone levels in PMOS. Furthermore, features derived from arHMM phase assignments show the potential for non-invasive PMOS screening using machine learning classification.

## Author summary

Tracking menstrual cycle information has become increasingly common, thanks in part to the availability of at-home urinary hormone data tracking technologies. In this study, we leveraged data from more than 3,800 menstrual cycles contributed by over 1,100 individuals in order to develop a model that predicts physiologically meaningful cycle phases based on day-to-day hormone patterns, exploring the potential of this model to provide ovulation proxies, improve clinical testing, and screen for reproductive disorders such as PMOS, a common endocrine disorder affecting approximately 1 in 10 reproductive age women. This study demonstrates the power of physiologically informed probabilistic modeling for leveraging data from at-home hormone monitoring towards a better characterization of menstrual cycle physiology and the development of noninvasive approaches for identifying reproductive health conditions such as PMOS.

## Introduction

Hormonal patterns across the menstrual cycle reflect women’s reproductive and overall health [1], and recent technological advances have created new opportunities to characterize these patterns at both the individual and population levels. Until recently, it was neither feasible nor cost-effective to track quantitative hormone patterns across the menstrual cycle, but at-home monitors that quantitatively measure urinary reproductive hormones have expanded our ability to non-invasively track menstrual hormonal trajectories [2]. Urine hormone testing is a convenient and relatively low-cost way to measure reproductive hormone levels. Hormone metabolites excreted in the urine accurately reflect hormonal patterns underlying menstrual cycle physiology [3,4]. With appropriate analysis frameworks, these self-tracked menstrual cycle data can be leveraged for improved population-level understanding of variability within and between women, and for learning novel hormonal biomarkers associated with reproductive disorders, including polyendocrine metabolic ovarian syndrome (PMOS), which affects approximately 10% of reproductive-age women and causes significant disease burden [5]. These frameworks may also be used to improve personalized insight into reproductive health, including ovulation confirmation, improved timing of clinical testing, and early detection of common menstrual-related disorders [6,7].

Within and between healthy women, there is considerable variation in hormone levels across the menstrual cycle, and in the timing of ovulation and cycle phases [8,9]. A meaningful model of the menstrual cycle must therefore account for this variability while also learning consistent patterns that govern hormone dynamics over the course of the cycle. Hidden Markov Models (HMMs), statistical frameworks widely used to model biological sequence data, are appropriate for labeling self-tracked time series due to their ability to describe complex relationships between observable and hidden biological states (i.e., phases of the menstrual cycle) [10–12]. Several studies have utilized HMMs to estimate ovulation timing and menstrual cycle phase with self-tracked data, including cervical fluid observations and basal body temperature [11,12]. To date, the HMM framework has not been used to predict menstrual cycle phase using self-tracked urinary reproductive hormone measurements, which may offer a more precise approximation of menstrual cycle physiology [3]. By defining phases probabilistically, the HMM approach aligns more closely with physiological processes that vary naturally across women and over time. This flexibility allows for accurate modeling of hormonal fluctuations regardless of menstrual cycle length or baseline hormone levels.

In this study, we use auto-regressive Hidden Markov Models (arHMMs) to label menstrual hormonal trajectories utilizing self-tracked urinary reproductive hormone measurements. This auto-regressive framework offers one potential approach for modeling menstrual cycle phases from quantitative urinary hormone data by capturing the dynamic nature of hormonal changes over time rather than relying on absolute hormone measurements. *The purpose of this study is to evaluate the suitability of arHMMs for this application.* Unlike traditional HMMs, which assume that observations are conditionally independent of previous observations given the current state, arHMMs incorporate the temporal dependence between successive hormone measurements. This allows the model to characterize the trajectories and rates of change in hormone concentrations that define the progression of ovarian-menstrual cycle physiology [13]. Instead of relying on absolute hormone values, our arHMM identifies latent menstrual phases based on the overall patterns and dynamics of hormone fluctuations, making it more robust to the substantial inter-individual variability observed in urinary reproductive hormone concentrations. By emphasizing hormonal trajectories rather than fixed thresholds, arHMMs may provide a more physiologically meaningful framework for identifying menstrual cycle phases and have the potential to improve the characterization of normal and abnormal reproductive physiology across diverse populations. *To demonstrate this potential, we use features derived from the arHMM to explore hormonal differences in women with and without self-reported PMOS and implement a proof-of-concept classifier to predict PMOS status.*

## Materials and methods

### Ethics statement

This study (protocol 2023/10/11) was approved by the Institutional Review Board at Duquesne University as Exempt according to 45CFR46.101(b)(4): (4) Secondary Research Uses of Data or Specimens on 10/21/2023. The data was stored by Mira according to the consent given by users in the data use agreement.

This was a secondary data analysis of urinary hormone data prospectively collected using the Mira Monitor (Quanovate Tech, Pleasanton, CA) in women with and without self-reported PMOS. Data included quantitative urinary reproductive hormone levels (estrone-3-glucuronide, E_1_3G, luteinizing hormone, LH and pregnanediol glucuronide, PDG), self-reported demographic information (date of birth), and self-reported health conditions (hormone imbalance, irregular cycle, PMOS, miscarriage history, recent birth control use, breastfeeding, postpartum not breastfeeding).

### Data collection

Urinary hormone data was collected using the Mira monitor, an at-home hormone analyzer that syncs with the Mira App (Fig 1). The Mira monitor is an FDA and CE-registered fertility tracker used to measure urinary estrogen (E_1_3G), luteinizing hormone (LH) and urinary progesterone (PDG). The Mira monitor has been validated for ovulation prediction (LH; R² = 0.96) and confirmation (R² = 0.86), demonstrating excellent agreement between quantitative urine hormone concentrations and ultrasound-confirmed ovulation [14].

**Fig 1:**
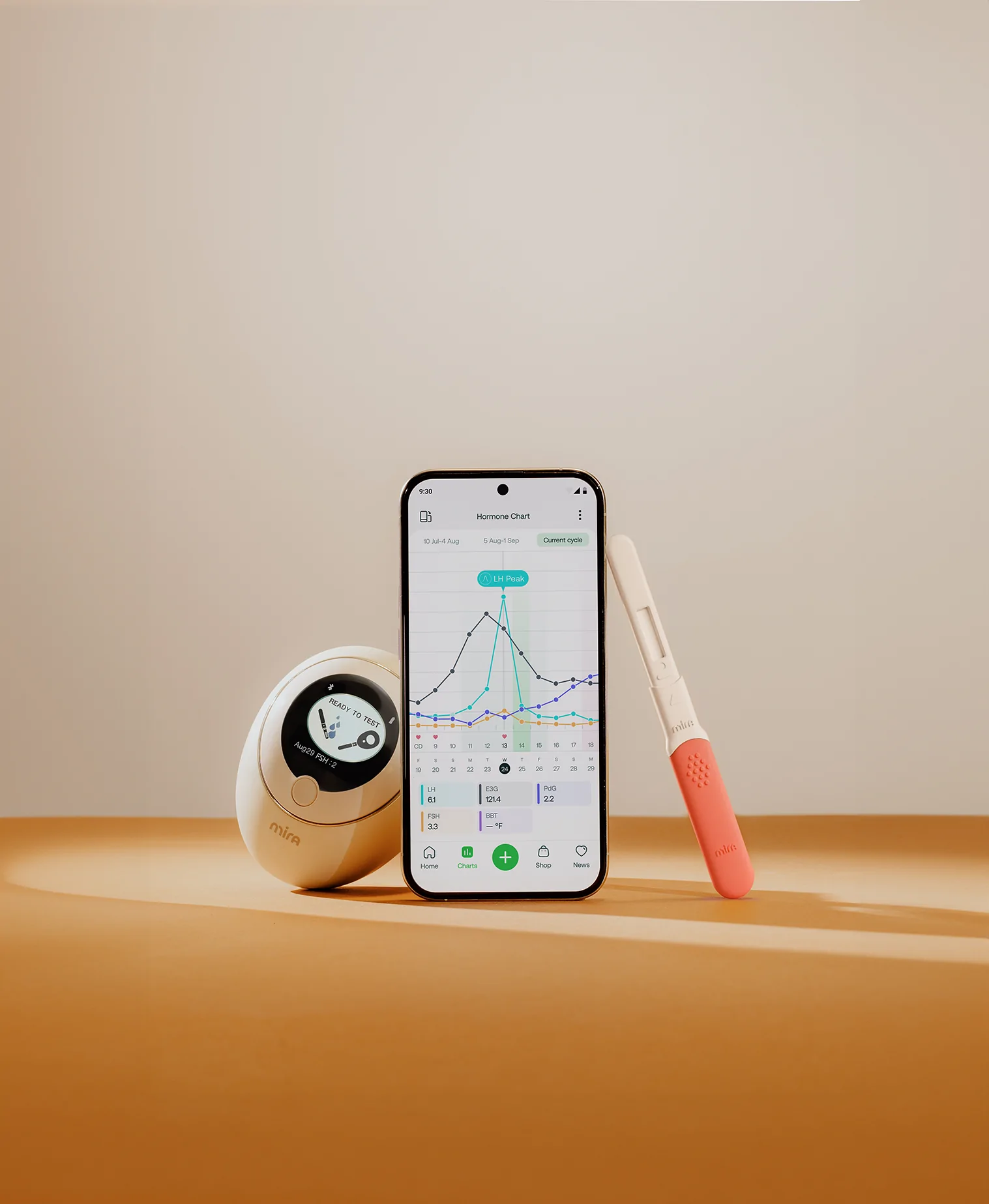
The Mira monitor, an at-home hormone analyzer that syncs with the Mira App.

Mira test wands detect urinary E_1_3G, LH and PDG levels based on fluorescence immunochromatography. The Mira monitor recognizes the test fluorescence signal and quantitatively converts it to the concentration of E_1_3G, LH and PDG. The user performs a test by dipping a Mira test wand into a urine sample for about 15 seconds and inserting it into the monitor. The results appear on the monitor in 21 minutes and can be synced to the Mira app through Bluetooth when paired with the monitor. The dataset included time-stamped urine hormone test results of E_1_3G, LH and PDG across recorded cycles.

The total dataset included 2,000 Mira users with at least three cycles of data, 1,000 Mira users with self-reported PMOS and 1,000 Mira users without self-reported PMOS. For all users, we removed cycles longer than 100 days as well as cycles recorded for users over the age of 45. We also implemented a filter to remove cycles without sufficient data using model-based criteria described below. To define each group, the clinical characteristics available in the dataset were limited to the following self-reported categories: hormone imbalance, irregular cycle, PMOS, miscarriage history, recent hormonal contraceptive use, breastfeeding, and postpartum not breastfeeding. Consequently, we were unable to account for the full range of clinical conditions and covariates known to influence menstrual hormone patterns. The inclusion criteria for healthy controls were women between the ages of 18–45. Exclusion criteria were any self-reported conditions other than recently coming off birth control and miscarriage history. The inclusion criteria for participants with PMOS were women between the ages of 18–45 and self-reported PMOS. Exclusion criteria for all participants was self-reported breastfeeding. For subgroup analyses based on cycle lengths (cycle frequency), participants were defined as having normal cycle frequency if at least 90% of their cycles were between 24–38 days [17]. Women with PMOS often have excessively long (> 38 days) and irregular menstrual cycles [18]. Since cycle length was the only phenotypic feature available in this dataset, we performed subgroup analyses to determine whether there were hormonal differences in healthy controls and women with self-reported PMOS based on cycle length.

The dataset included the following cycle-level information: cycle length, cycle start date, and cycle end date as well as all time-stamped urine hormone readings collected on a given cycle day. If a cycle day included more than one test result, we took the first recorded level of E_1_3G and PDG and the maximum LH level recorded that day. We aligned the cycle and hormone data in order to assign each hormone measurement to the corresponding day of the cycle (with day 1 representing the first day of bleeding), allowing for the visualization of single cycle patterns within and across individuals (“forward indexing”).

In order to account for variation in cycle length, and the increased variation of the follicular phase relative to the luteal phase [10], we also implemented two additional indexing strategies: 1) alignment to the end of the cycle (“backward indexing”), in which day -1 corresponds to the last day of the cycle, and 2) alignment to the LH surge (“LH-indexing”), in which day 0 corresponds to the day with the highest recorded LH value across that cycle, day -1 corresponds to the day prior to the LH surge, and day 1 corresponds to the day following the LH surge. These three indexing strategies allowed for the analysis and visualization of average hormonal trends within our healthy controls, with the average hormone patterns in the follicular phase, luteal phase, and around the LH surge brought into focus using forward-, backward- and LH-indexing respectively.

### Learning auto-regressive hormone patterns and building the autoregressive Hidden Markov model (arHMM)

To develop a “typical” model of day-to-day hormone patterns over the course of a single menstrual cycle, we first curated a set of cycles belonging to our healthy control population, filtering for cycles that were between 24 and 38 days long [17], for which missingness was less than 50% (i.e., the number of recordings divided by the cycle length was at least 50%, indicating testing on average at least every other day). For our model comparison and random forest classifier analysis, we further restricted this set, using only 20% of the curated set of cycles and setting aside the remaining 80% for classifier building and testing. We then divided the menstrual cycle into six general phases based on observed average hormone patterns using the three cycle indexing strategies described above, which together provided clearer patterns at the beginning and end of the cycle, as well as around the LH surge. These six phases are defined by distinct hormonal trajectories during which E_1_3G, LH, and PDG exhibit relatively consistent patterns of rising, falling, or stable concentrations. This six-phase framework [15] aligns closely with ovarian-menstrual physiology and its reliance on trajectories fits well within the arHMM framework, which leverages temporal changes in hormone concentrations to identify latent ovarian-menstrual states rather than relying on fixed hormone thresholds [13]. In our curated cycles, these phases approximately corresponded to: the early follicular phase (the first 5 days of the cycle; phase 1), the late follicular phase characterized by a rise in E_1_3G (10–2 days prior to the LH surge; phase 2), the peri-ovulatory phase marked by rising E_1_3G and LH levels (2–0 days before the LH surge; phase 3), the early luteal phase with increasing PDG production (0–4 days after the LH surge; phase 4), the mid-luteal phase with relatively stable E_1_3G and PDG levels (9–5 days before the end of the cycle; phase 5), and the late luteal phase characterized by declining E_1_3G and PDG levels (the final 5 days of the cycle; phase 6). The ranges used to define the arHMM phases establish the model’s parameters, but they do not fix the duration of each phase. The exact amount of time spent in any given phase is expected to vary across cycles and individuals, consistent with the natural variability in hormonal timing described above.

For each phase, and for each of the three hormones, we built a simple linear regression model to predict the hormone level on day i + 1 given the hormone level observed on day i, using all recorded consecutive days for which the second day belonged to the phase of interest (as specified in the previous paragraph). In order to enforce linear trajectories over multiple days, we set the slope to 1, and estimated the intercept ($b_{j}^{k}$) and variance ($\sigma_{j}^{\text{2}k}$) for each hormone k and phase j = 1,...,6. We note that a positive intercept results in a positive linear trend over days, while a negative intercept results in a negative linear trend over days; this model is akin to a random walk with a phase-specific drift term. In practice, this captures the general behavior of each hormone in each phase: e.g., PDG tends to rise in phase 4 (positive intercept), while LH tends to fall (negative intercept), while allowing the absolute hormone levels on a given day to be variable within and between individuals. Given the exploratory nature of this study, we chose linear trajectories as a parsimonious and interpretable framework for characterizing hormonal changes within each phase of the menstrual cycle.

The six cycle phases described above constitute the latent states in our autoregressive Hidden Markov model (arHMM), which are assigned to cycle days using a viterbi algorithm. Since our primary goal is to model the temporal progression of hormonal patterns across a healthy ovarian-menstrual cycle, we use a transition matrix that ensures that each cycle must progress through each phase in order, while allowing the number of days spent in each phase to vary. We set transition probabilities based on the observed number of days spent in each phase on average in our curated set of cycles (Table A in S1 Appendix). For this study, we constrained the transition matrix as a starting point for examining whether arHMMs can recover physiologically meaningful cycle trajectories from quantitative urinary hormone data. Given the real-world nature of the data, imposing sequential transitions leverages established knowledge of ovarian physiology and provides an initial framework for exploring differences between groups based on limited self-reported clinical characteristics. Although this constrained framework is well suited for identifying physiologically meaningful phases, more flexible transition matrices may ultimately be needed to improve classification of individuals with PMOS, in which hormonal trajectories may deviate as a result of underlying endocrine disruption [16].

The autoregressive piece of the arHMM comes from the emission model, which models the emission for a hormone k on day i, $X_{i}^{k}$ as a stochastic linear function of the latent state (phase) on day i ($Y_{i})$ and the hormone level on day i-1:

$e_{i}^{k}\;=f_{X_{i}^{k}}(x_{i}^{k}|Y_{i}=j,\;X_{i-\text{1}}^{k}=x_{i-\text{1}}^{k}\;)=\;x_{i-\text{1}}^{k}+b_{j}^{k}+\epsilon$

where $\epsilon \sim Normal(\text{0},\;\sigma_{j}^{\text{2}k}\;)$, and intercept $b_{j}^{k}$ and variance $\sigma_{j}^{\text{2}k}$ are the phase- and hormone-dependent constants learned from our data as described above (Table B in S1 Appendix). We make a simplifying assumption that the three hormones are conditionally independent given the latent states, such that the complete emission on day i is given by $e_{i}=e_{i}^{E\text{3}G}e_{i}^{LH}e_{i}^{PDG}$.

To produce phase predictions for a cycle, that cycle must have exactly one entry each of PDG, E_1_3G, and LH for each day of the cycle. This required both imputing values for days on which the individual did not record their hormone data, and also reducing the amount of data on days when the individual recorded multiple times (as described above). To impute missing data, we used a linear interpolation approach: for any span of days with missing data, we took the nearest flanking hormone readings and assumed a linear trend in between. For missing data before the first recorded day, the hormone value was set to the value of the first available recording. For missing data after the last recorded day, the hormone value was set to the last available recording. Because of the importance of particular hormones in different phases of the menstrual cycle and the difficulty of linear interpolation in these phases, we restricted all analyses to individuals with E_1_3G readings on at least 50% of phase 2 and phase 3 days, LH readings on at least 50% of phase 3 and phase 4 days, and PDG readings on at least 50% of phase 4 and phase 5 days. This missing-data filtering was explicitly incorporated to maximize available data while removing cycles that did not have sufficient observations during key hormonal inflection points.

### Model comparison

We compared the arHMM model performance to that of a simple Naive Bayes classifier using the limited ground truth information available in this dataset. This Naive Bayes classifier independently classifies each cycle day as belonging to the follicular or luteal phase without access to the correlations present in day-to-day hormonal trajectories. To approximate ground truth, we used the maximum recorded urinary LH value as a proxy measure for identifying the follicular and luteal phases. Specifically, the day of the maximum recorded LH value was designated as the final day of the follicular phase, with all subsequent days classified as the luteal phase. Because hormone measurements were not necessarily collected daily, the observed maximum LH value may not always capture the true LH surge, and this approach may mis-label phases in anovulatory cycles or cycles characterized by atypical LH patterns. To minimize these limitations in our model comparison, we trained and tested both models on the curated cycles described above.

For the arHMM-based classifier, the six latent menstrual-cycle phases were collapsed into the biphasic follicular/luteal categories for comparison with the reference labels. In accordance with how our six phases are defined in alignment with underlying menstrual cycle physiology, days assigned by the arHMM to phases 1–3 were classified as follicular, whereas days assigned to phases 4–6 were classified as luteal [17].

The Naive Bayes classifier was trained on distributions learned from the labeled training data using the GaussianNB implementation in Python’s scikit-learn package with equal (50:50) prior probabilities for the follicular and luteal phases. Model performance was evaluated using a receiver operating characteristic (ROC) curve, enabling comparison of the sensitivity and specificity of the arHMM model in the same space.

### Exploring clinical applications

To compare hormone testing protocols using fixed cycle days vs arHMM-derived phases, we focused in particular on LH in phase 3 and on days 13–16 and on PDG in phase 5 and on days 19–22. For LH, we calculated the maximum hormone level for each cycle within the day or phase window, and for PDG, we calculated the average hormone level for each cycle within the day or phase window. We used paired t-tests to evaluate whether the hormone levels differed when considering phases vs days within each comparison.

To examine differences between healthy controls and women with self-reported PMOS, we developed individual-level features based on arHMM output:

#### Hormone features.

Within each cycle, for each hormone, and each HMM-predicted phase [1–6], we extracted the minimum, maximum, mean, median, and standard deviation of the hormone values that fall within that phase. This resulted in 90 cycle-level features. To construct individual-level features, we took the minimum, maximum, mean, median, and standard deviation of each cycle-level feature over all cycles the individual had recorded, yielding 450 individual-level features.

#### Phase length features.

Within each cycle, we extracted the number of days spent in each of the six HMM-predicted phases, resulting in 6 cycle-level features. We then calculated the minimum, maximum, mean, median, and standard deviation for each phase length across all cycles the individual has recorded, yielding 30 individual-level features.

#### Model fit features.

One output of the HMM is the joint log-probability of the entire sequence of hormones for a given cycle together with the maximum likelihood path, which can be interpreted as a measure of how well the cycle fit the model. From this single cycle-level feature, we generated five individual-level features by taking the minimum, maximum, mean, median, and standard deviation of the log-probability scores across all cycles recorded by an individual.

We constructed a random forest classifier using python library scikit-learn based on the arHMM-derived features described above [18], training the classifier to distinguish between individuals with PMOS from healthy controls. We also trained a second classifier that adds non-arHMM-derived cycle length features (mean, minimum, maximum, median, standard deviation, and range across all cycle lengths for the individual). The random forest classifier was implemented with 500 trees and a max depth of 10, though setting the classifier to use default parameters (100 trees and no max depth) did not appreciably change the performance. We balanced our dataset to include equal numbers of individuals with and without self-reported PMOS and used a 25/75 stratified train/test split. Accuracy was calculated as the fraction of correct predictions pooled across true PMOS and healthy control individuals, and we calculated sensitivity as the fraction of individuals with PMOS who were correctly classified, specificity as the fraction of healthy control individuals who were correctly classified, positive predictive value as the fraction of PMOS classifications that were correctly assigned, and negative predictive value as the fraction of healthy control classifications that were correctly assigned. Reported rates are averaged over 100 random train-test splits.

To label a cycle as ovulatory versus anovulatory, we built a proxy measure based on PDG levels in phase 5 (representing the post-ovulatory rise in progesterone) [14]. Average PDG levels in phase 1 and 2 for the same cycle are used as baseline PDG levels. For a cycle to be labeled ovulatory, we required a number of days (*n*) in phase 5 in which PDG levels were higher than the phase 1–2 baseline by a specific threshold. We considered thresholds ranging from a minimum of a 25% increase over baseline up to a maximum threshold of a 200% increase over baseline, in increments of 25%. We considered a requirement of *n* = 1, 2, or 3 days. For cycles that did not have at least *n* PDG readings in phase 5, or had no recorded PDG readings in phases 1 or 2, we did not label the cycle as ovulatory or anovulatory. The remaining cycles were labeled anovulatory.

## Results

### Descriptive analyses

This secondary analysis included 1,269 cycles from 277 healthy controls and 2,563 cycles from 841 women with self-reported PMOS (after applying inclusion and exclusion criteria described above in methods). Examples of two recorded cycles are shown in Fig 2A, one for a healthy control individual, and one for an individual with self-reported PMOS. Table 1 includes mean ages at the time of recording cycle data, mean cycle lengths, and mean number of cycles recorded for healthy controls and women with self-reported PMOS. On average, women with self-reported PMOS had longer and more irregular cycles as expected based on the clinical characteristics of this condition (Fig 2B).

**Table 1:** Study population characteristics. Age is recorded in years, and cycle length is recorded in days.

Variable	Healthy Controls (n = 277)	PMOS (n = 841)
Number of Cycles	1269	2563
Mean (SD) Age at Time of Recording Cycle	36.3 (4.79)	34.05 (4.44)
Mean (SD) Cycle Length	28.55 (5.21)	32.05 (7.55)
Mean (SD) Number of Cycles Recorded per Individual	24.89 (11.28)	20.11 (11.03)
% of Cycles Between 24–38 Days	91%	82%

**Fig 2:**
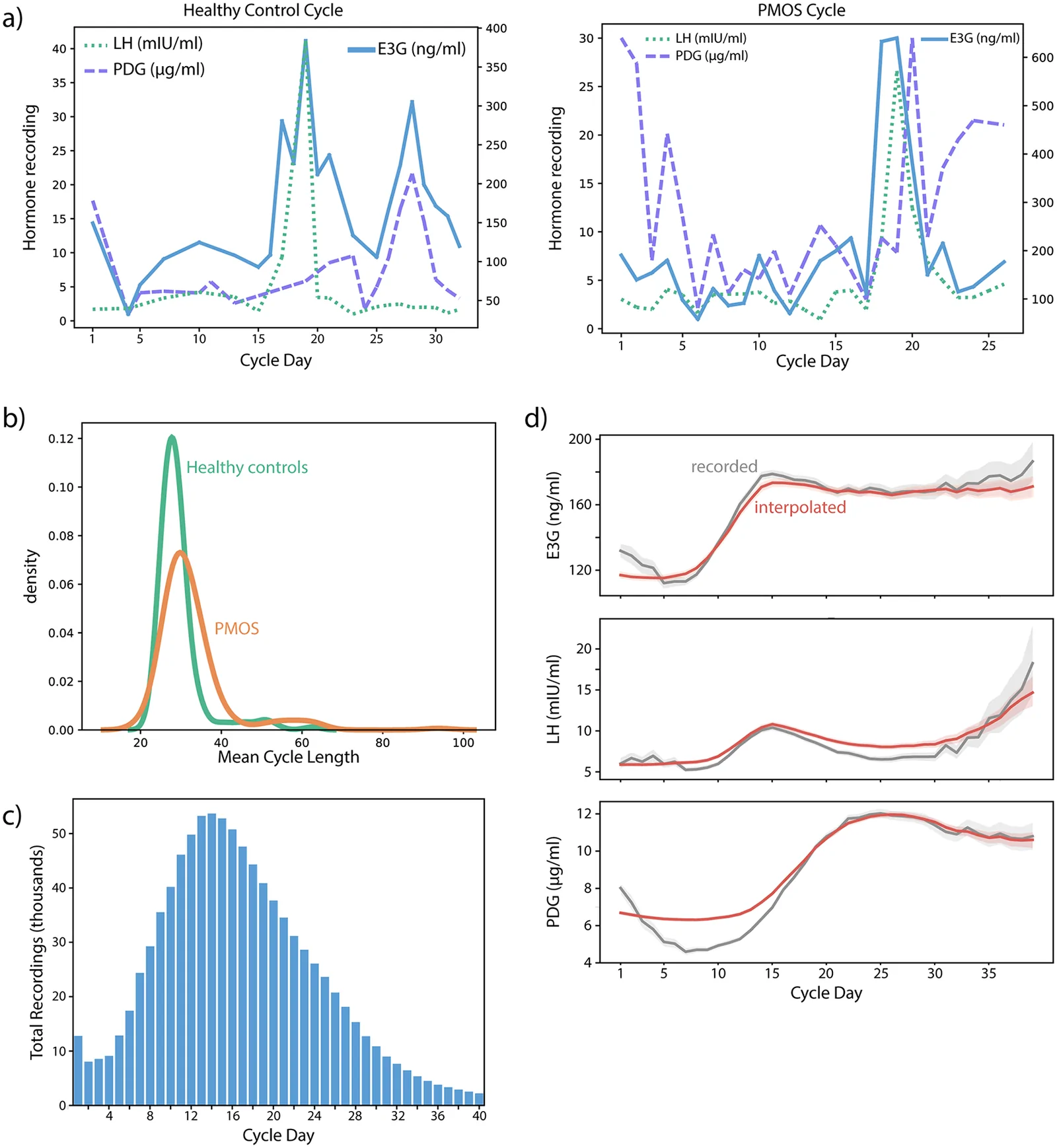
**A) Single cycle data**. LH, PDG, and E_1_3G recordings for two cycles, one belonging to a healthy control individual (left) and one belonging to an individual with self-reported PMOS (right). Hormone values for LH and PDG are on the left vertical axis, and values for E_1_3G are on the right vertical axis. **B) Distribution of cycle lengths.** Cycle lengths for healthy control cycles and PMOS cycles have overlapping distributions, but PMOS cycles were on average longer (mean of 28.55 days versus 32.05 days; p < 0.001). **C) Recording behavior as a function of cycle day**. Individuals were much more likely to take hormone measurements towards the middle of the cycle, and there was a large amount of missing data at the beginning and end of the cycle. **D) Performance of missing data imputation**. In light of the missing data illustrated in panel C, we imputed recordings on missing days using the interpolation approach described in Methods. Gray lines represent available measurements for each hormone across cycle day, and red lines represent all measurements after interpolation. The interpolated values did not deviate dramatically from the available data, especially in realms where each hormone is dominant (E_1_3G in the luteal phase, LH around ovulation, and PDG in the follicular phase), though the approach did tend to overestimate PDG in the follicular phase.

Hormone testing behavior was not uniform over the course of the menstrual cycle, with tests increasing towards the middle of recorded cycles around the presumed LH surge and ovulation (Fig 2C). Our analyses required imputation of missing data as described in Methods; we found that the imputed values tracked well with the available hormone levels in the dataset (Fig 2D). In particular, E_1_3G tracked well in the follicular phase when it is expected to be dominant, LH tracked well near the periovulatory window, and PDG tracked well in the luteal phase when it is expected to be dominant.

Absolute hormone levels across the menstrual cycle vary widely between individuals [8], which makes modeling these levels difficult with a standard HMM approach in which the model learns distributions for the absolute levels of each hormone throughout the cycle. To better suit this data and the way in which we expected hormone *trajectories* to shift over the different cycle phases, we implemented an autoregressive HMM (arHMM) which instead models the hormone levels as time series, with phases in which hormones are expected to rise, fall, or remain constant. As a result, two cycles with very different absolute hormone levels, but similar directional trajectories would be fit equally well by the model.

We used the three indexing strategies described in Methods to map hormone levels to six cycle phases (1 = early follicular, 2 = late follicular, 3 = periovulatory, 4 = early luteal, 5 = mid-luteal, and 6 = late luteal) (Fig 3A-3C).

**Fig 3:**
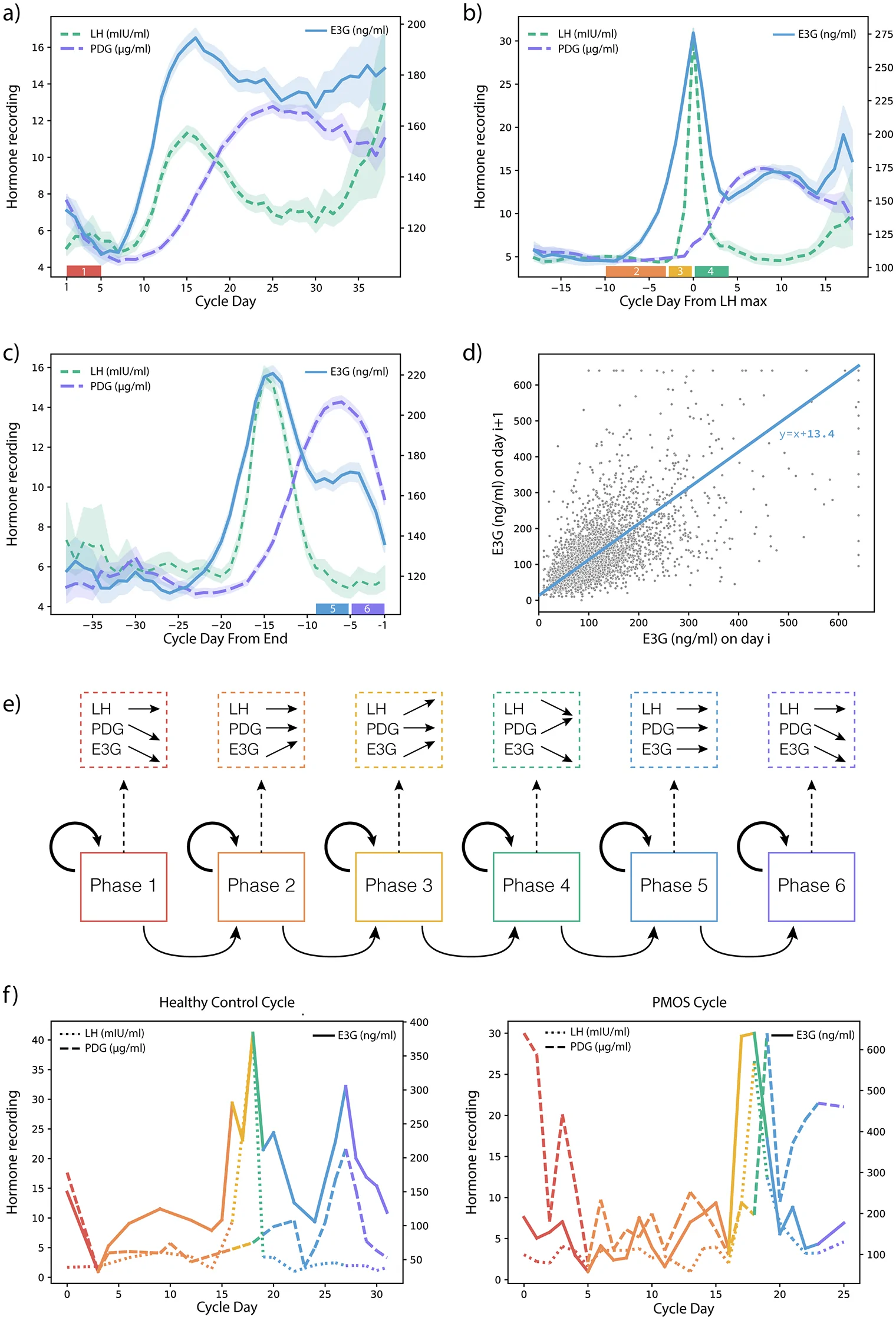
**A-C) Forward, LH, and Backward indexing in cycles used for arHMM training.** Forward indexing marks the first day of bleeding as day 1; this is used to learn trajectories in phase 1 (early follicular). LH indexing marks the day on which LH reaches a maximum as day 0; this is used to learn trajectories in phase 2 (late follicular), phase 3 (periovulatory), and phase 4 (early luteal). Backward indexing marks the last day of the cycle as day -1; this is used to learn trajectories in phase 5 (mid-luteal) and phase 6 (late luteal). **D) Learning phase trajectories.** E_1_3G hormone measurements are graphed for each pair of consecutive days in phase 2 within the curated set of cycles used for training the arHMM. In blue is the best fit line with a fixed slope of 1; within phase 2, we model E_1_3G rising by 13.4 ng/ml each day on average. **E) Schematic of arHMM.** Solid boxes outline the six hidden states of the arHMM, phases 1-6. Solid lines represent transition probabilities; paths through the model must start at phase 1 and end at phase 6 and can remain within phases for multiple days. Dotted boxes and lines represent the autoregressive emission model; within each phase, each hormone has an expected linear trajectory day over day (see Fig 3D). Briefly, PDG and E_1_3G are expected to fall in phase 1, E_1_3G rises in phase 2, E_1_3G and LH rise in phase 3, LH and E_1_3G fall in phase 4 while PDG rises, and PDG and E_1_3G fall in phase 6. **F) Example arHMM phase assignments.** For the same cycles as in Fig 2A, the arHMM viterbi path predicts phases for each day of the cycle, beginning in phase 1 (red), then moving in sequence through phase 2 (orange), phase 3 (yellow), phase 4 (green), phase 5 (blue), phase 6 (purple).

To develop the arHMM, we used a curated set of 626 cycles from 189 healthy controls as described above, using these cycles to learn day-to-day trajectories for each hormone in each phase (Fig 3D and Table A in S1 Appendix). The full schematic of the arHMM is included in Fig 3E. The arHMM produces phase predictions for each cycle in the dataset (total cycles in each group included in Table 1); phase assignments for two example cycles are provided in Fig 3F.

We found that the average hormone levels within assigned phases across all cycles were consistent with expected menstrual cycle hormonal trajectories: E_1_3G rises in phase 2 and phase 3, LH surges in phase 3, and PDG rises in phases 4 and 5 (Fig 4A and Fig A in S1 Appendix).

**Fig 4:**
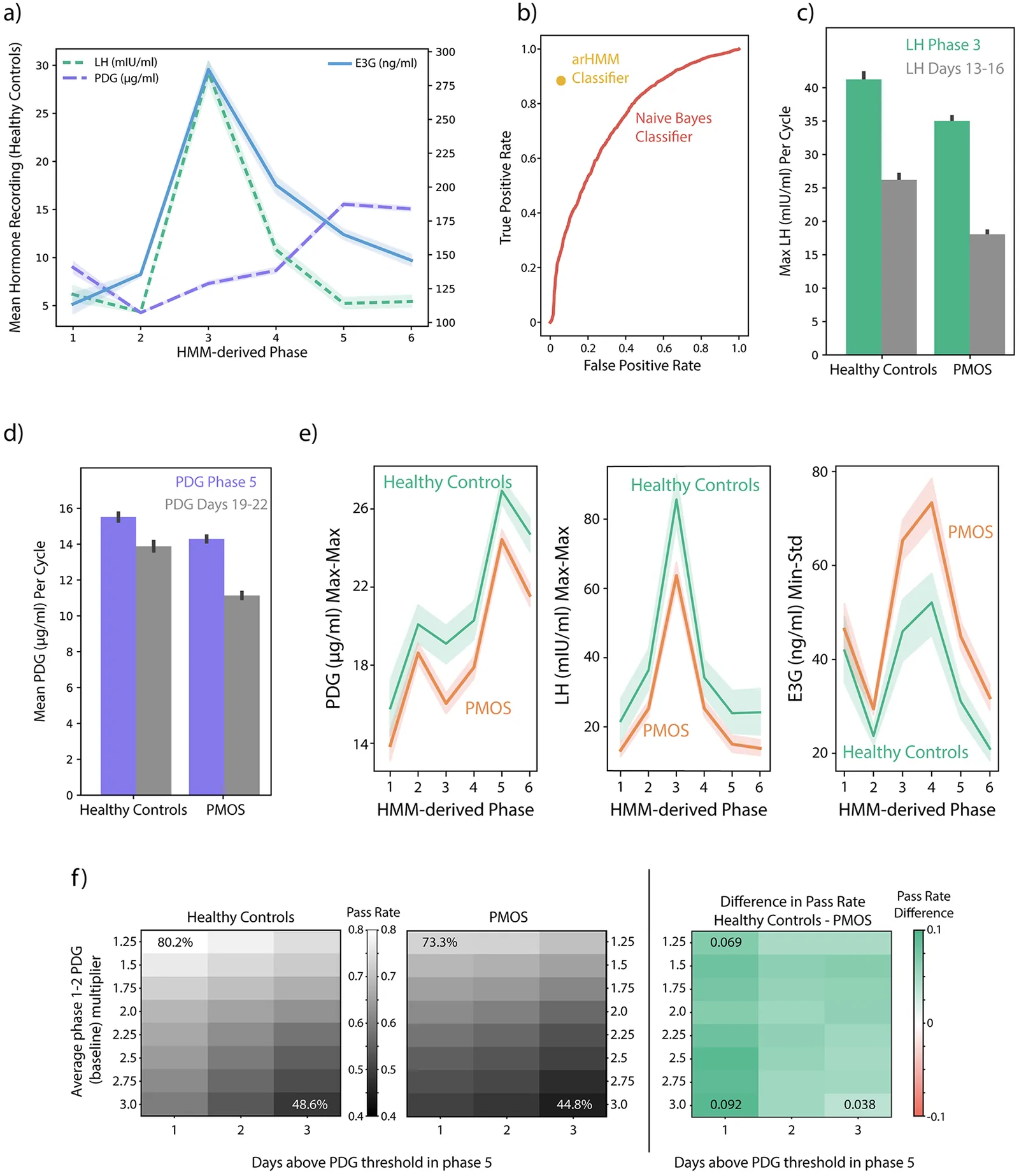
**A) Average hormone levels in each arHMM-derived phase**. Averaging over all healthy control cycles, and all recordings per phase within each cycle, the arHMM reproduced expected patterns: a rise in E_1_3G in phase 2 (late follicular) and phase 3 (ovulation), a rise in LH in phase 3 and subsequent fall in phase 4 (early luteal), and a rise in PDG in phase 5 (mid-luteal). **B) Learning trajectories with the arHMM improves classification of follicular and luteal phases.** We labeled cycle days as follicular (0) or luteal (1) based on their position relative to the day of maximum recorded LH, and trained a Naive Bayes classifier to make predictions based on LH, E_1_3G, and PDG levels on a given day. False positive rate (1-specificity) represents the proportion of follicular cycle days that are misclassified as luteal, and true positive rate (sensitivity) represents the proportion of luteal cycle days that are correctly classified as luteal. We produce the ROC curve by varying the threshold used to binarize the probability output of the Naive Bayes classifier. For the arHMM classifier, we can only report a single false positive rate/true positive rate pair, which nonetheless outperforms any choice of threshold for the Naive Bayes classifier. **C) LH surges were significantly more robust in phase 3 than on days 13-16 of the menstrual cycle.** For each cycle, we calculated the maximum LH value observed in phase 3 and the maximum LH value observed across days 13-16. While PMOS cycles had lower LH values than healthy control cycles, within both groups, a cycle-day testing protocol is less effective at recording the LH surge than testing during phase 3. **D) PDG rises were significantly more robust in phase 5 than on days 19-22 of the menstrual cycle.** For each cycle, we calculated the mean PDG value observed in phase 5 and the mean observed across days 19-22. We saw a similar result; PMOS cycles have lower PDG levels than healthy control cycles on average, but within both groups, the cycle-day testing protocol is less effective at recording the luteal phase PDG rise. **E) arHMM-derived hormone features reveal differences in PMOS and healthy control individuals.** We calculated individual-level features as described in Methods; these aggregated hormone recordings across all available cycle days within a particular phase, followed by an aggregation across all cycles for an individual. Three examples of these individual level features are shown here, across all six phases. Left: Maximum PDG within each phase, maximized across all cycles; Middle: Maximum LH within each phase, maximized across all cycles; Right: Minimum E_1_3G within each phase, with standard deviation across all cycles. The latter reflects an increase in variability cycle-to-cycle within PMOS individuals. **F) Self-reported PMOS cycles were less likely to pass threshold-based ovulation tests.** Left (healthy controls) and middle (PMOS): heatmaps show the fraction of cycles that were labeled ovulatory for pairs of threshold parameters: a number of days (columns) with PDG levels over a particular value (rows; as a multiplier of average phase 1 and 2 PDG levels). Right: difference in pass rates, healthy controls - PMOS. Across the choices of threshold parameters, PMOS cycles were less likely to be labeled as ovulatory.

### Model comparison

The Naive Bayes classifier achieved an area under the ROC curve of 0.748, indicating discrimination better than chance but with a substantial degree of misclassification. Because the arHMM-based classifier does not produce a variable classification threshold, its performance is not readily represented by a conventional ROC curve. Instead, its performance can be represented as a single operating point in ROC space, with a false-positive rate of 0.057 (specificity of 0.943) and a true-positive (sensitivity) rate of 0.884 (Fig 4B). This operating point indicates substantially better classification performance than the Naive Bayes classifier.

### Clinical applications of arHMM-derived phases

Clinicians order laboratory testing on specific days of the menstrual cycle to evaluate patients for underlying menstrual-hormonal abnormalities or to schedule ultrasounds for ovulation confirmation [19]. For example, to evaluate periovulatory hormone patterns, laboratory testing may be ordered on cycle days 13–16. To explore differences in hormone concentrations using arHMM-derived phases rather than fixed cycle days, we compared maximum LH levels in phase 3 to maximum LH levels on days 13–16. Hormonal testing is also frequently ordered on cycle days 19–22 to examine the post-ovulatory rise in progesterone as a useful proxy measure for ovulation [20]. We also compared PDG levels in phase 5 to mean PDG on days 19–22, as described in Methods. In both healthy controls and individuals with PMOS, we found that the phase-based levels are significantly higher than the cycle-day-based levels (p < 0.001 for all four comparisons; Fig 4C-4D). These differences are expected, given both how the arHMM was trained, defining phases according to specific hormonal trajectories, and the well-established variability in the timing of these trajectories within and between individuals [9]. This supports phase-based testing as one promising approach for obtaining accurate hormone measurements independent of specific cycle days.

Table 2 contains average cycle-level features (described in Methods) calculated within healthy control cycles and within PMOS cycles. We find that in many instances, hormone features within PMOS cycles differ significantly from those within healthy control cycles, including higher E_1_3G in phases 3, 4, and 5, lower LH in phase 3, and lower PDG in phases 5 and 6. In addition to analyzing mean hormone levels within phases, we also considered minimum and maximum levels within each phase, as a mechanism for examining potential differences in the hormone ranges (e.g., maximum LH in phase 3 tended to be higher in healthy controls compared to individuals with PMOS). We also found that the length of time spent in some phases differed significantly (Table 3); in particular, PMOS cycles spent a significantly longer time in phase 2 than healthy control cycles (p < 0.001), which is consistent with the pathophysiology of PMOS, characterized by oligomenorrhea and ovulatory dysfunction [21]. We note that because of the prevalence of missing data at the start of the cycle (Fig 1C), the arHMM has a difficult time distinguishing between phase 1 and phase 2, which together estimate the length of the follicular phase prior to ovulation (about 14 days for healthy control cycles). This results in the model transitioning to phase 2 very quickly in most cases, which accounts for the shorter reported phase 1 durations and long reported phase 2 durations in both PMOS and healthy control cycles. We also note that phase 4 durations appear shorter than expected based on our training; we speculate that this is due to differences among our curated set of healthy controls used for training and the larger set of cycles that include different patterns of missingness and cycle lengths. We also examined differences between subgroups based on cycle length (reported in Table 3 and Tables C and D in S1 Appendix). Overall, when grouped by cycle length (e.g., normal cycle length healthy controls vs normal cycle length PMOS) we still identified distinct hormonal patterns within the arHMM-derived cycle phases.

**Table 2:** For each cycle, for each hormone and phase, we calculate the mean, minimum, and maximum for all available hormone measurements within that phase of the cycle (cycle-level features described in Methods). The table presents the averages and standard deviations of these cycle-level features separated into healthy control (HC) cycles and PMOS cycles. P-values are listed as not significant (ns), p < 0.05 (*), p < 0.01 (**), and p < 0.001 (***).

Phase	E3G HC (ng/ml)	E3G PMOS (ng/ml)	p-value	LH HC (mIU/ml)	LH PMOS (mIU/ml)	p-value	PDG HC (mg/ml)	PDG PMOS (mg/ml)	p-value
Phase 1 average mean (SD) [avg min (SD) – avg max (SD)]	**120.12 (106.13)** [107.89 (107.12)- 132.79 (110.66)]	**125.15 (96.34)** [115.58 (96)- 138.22 (107.6)]	ns ns ns	**5.47 (14.3)** [4.62 (14.13)- 6.66 (15.12)	**4.84 (5.27)** [4.01 (3.38)- 6.23 (12.13)]	ns ns ns	**7.78 (6.58)** [5.86 (5.84)- 10.25 (9.32)]	**7.23 (6.16)** [5.93 (5.6)- 9.0 (8.56)]	ns ns ns
Phase 2 average mean (SD) [avg min (SD) – avg max (SD)]	**139.52 (72.62)** [68.98 (48.81)- 244.99 (126.31)	**136.13 (80.27)** [63.78 (58.96)- 252.24 (138.99)]	ns ** ns	**4.59 (8.95)** [2.51 (7.62)- 8.05 (10.65)]	**4.85 (3.48)** [2.29 (2.12)- 9.18 (6.32)]	ns ns ***	**4.84 (4.43)** [2.91 (3.76)- 7.6 (5.96)]	**5.15 (4.24)** [2.93 (3.4)- 8.39 (5.95)]	ns ns ***
Phase 3 average mean (SD) [avg min (SD) – avg max (SD)]	**275.28 (152.13)** [195.11 (146.01)- 358.15 (191.87)	**281.12 (160.13)** [205.99 (153.02)- 360.26 (200.91)]	ns ** ns	**25.49 (17.98)** [13.79 (12.41)- 41.24 (34.42)]	**22.24 (16.64)** [13.06 (12.29)- 35.01 (31.6)]	*** ns ***	**7.25 (6.26)** [5.86 (6.02)- 8.76 (7.33)]	**7.67 (6.23)** [6.51 (6.09)- 9.94 (8.06)]	ns ** ns
Phase 4 average mean (SD) [avg min (SD) – avg max (SD)]	**185.21 (115.76)** [146.87 (98.6)- 224.88 (157)]	**195.08 (123.91)** [155.41 (105.97)- 236.53 (166.92)]	* * *	**9.53 (17.3)** [6.72 (16.86)- 12.97 (19.49)]	**9.4 (13.97)** [6.61 (13.02)- 12.79 (16.88)]	ns ns ns	**8.56 (7.08)** [7.42 (6.92)- 9.78 (7.94)]	**8.81 (7.21)** [7.73 (7.01)- 9.94 (8.06)]	ns ns ns
Phase 5 average mean (SD) [avg min (SD) – avg max (SD)]	**162.18 (105.66)** [126.83 (98.43)- 207.76 (138.37)]	**172.38 (114.55)** [138.31 (109.26)- 217.64 (149.15)]	* ** ns	**5.41 (17.05)** [4.43 (17.08)- 6.73 (17.22)]	**5.48 (12.58)** [4.41 (11.73)- 6.82 (13.11)]	ns ns ns	**15.52 (7.66)** [10.27 (8.3)- 21.33 (9.19)]	**14.29 (7.92)** [9.87 (8.24)-19.41 (9.81)]	*** ns ***
Phase 6 average mean (SD) [avg min (SD) – avg max (SD)]	**142.32 (102.62)** [112.53 (96.14)- 176.34 (121.99)]	**146.92 (101.95)** [119.6 (97.25)- 178.75 (121.68)]	ns ns ns	**4.78 (12.2)** [3.56 (11.64)- 6.8 (17.52)]	**5.26 (15.88)** [4.28 (15.84)- 6.61 (16.29)]	ns ns ns	**14.6 (8.38)** [10.88 (8.55)- 17.94 (9.4)]	**13.1 (8.48)** [9.95 (8.41)- 16.07 (9.42)]	*** ** ***

**Table 3:** The table presents the averages and standard deviations of the number of days spent in each phase separated into healthy control (HC) cycles and PMOS cycles and subgroups based on cycle length (normal cycle length vs. abnormal cycle length). P-values are listed as not significant (ns), p < 0.05 (*), p < 0.01 (**), and p < 0.001 (***).

arHMM-Phase	HC	PMOS	p-value	HC Normal Cycle Length	PMOS Normal Cycle Length	p-value	HC Abnormal Cycle Length	PMOS Abnormal Cycle Length	p-value
Phase 1	1.34 (1.75)	1.32 (1.98)	ns	1.23 (1.37)	1.27 (1.51)	ns	1.47 (2.11)	1.36 (2.23)	ns
Phase 2	13 (4.74)	16.87 (7.81)	***	13.07 (4.18)	14.4 (4.68)	***	12.92 (5.36)	18.44 (8.92)	***
Phase 3	1.82 (1.91)	1.74 (1.66)	ns	1.81 (1.76)	1.75 (1.53)	ns	1.82 (2.08)	1.73 (1.73)	ns
Phase 4	1.51 (0.76)	1.58 (1.11)	*	1.5 (0.71)	1.6 (1.05)	*	1.51 (0.83)	1.57 (1.14)	ns
Phase 5	3.63 (2.71)	3.56 (3.06)	ns	3.66 (2.69)	3.53 (2.78)	ns	3.6 (2.74)	3.58 (3.23)	ns
Phase 6	7.25 (4.64)	6.98 (4.5)	ns	7.38 (4.53)	7.11 (3.78)	ns	7.1 (4.78)	6.89 (4.91)	ns

As described in Methods, we also aggregated cycle-level features into individual-level features that incorporated the arHMM output across all recorded cycles for a given individual. Fig 4E illustrates some of these individual-level features: we found that the hormone levels for E_1_3G, LH, and PDG had distinct patterns across the six phases for individuals with self-reported PMOS compared to healthy control individuals.

To explore the potential of these features to detect PMOS in individuals, we implemented a random forest classifier as described in Methods, using the restricted arHMM training set to avoid overlap in arHMM training and classifier development that might otherwise lead to overfitting. When using balanced training and testing sets, we see an overall accuracy of 67% (68% sensitivity, 66% specificity, 67% positive predictive value, 67% negative predictive value). If we add cycle length features, overall accuracy increases to 70% (71% sensitivity, 68% specificity, 69% positive predictive value, 70% negative predictive value). We note that the ceiling on the performance of this classifier is limited both by a relatively small sample size for training after class balancing, as well as the use of self-reported PMOS status. With larger, validated datasets, it is possible that similar classifiers could achieve significantly better performance.

In examining the model fit features described in Methods, we observed that model fit declined as a function of cycle length, which is an expected pattern. When accounting for this relationship with cycle length, however, we do not see a difference in model fit between healthy control cycles and PMOS cycles (Fig B in S1 Appendix). This is likely a reflection of the flexibility of the arHMM itself, and its reliance on directional trajectories rather than absolute hormone levels; this suggests that the arHMM is able to effectively model most PMOS cycles along with healthy control cycles.

Previous studies suggest that a useful proxy measure of ovulation is an LH surge followed by elevated PDG levels for multiple days [20]. As described in Methods, we labeled a cycle as ovulatory versus anovulatory based on PDG levels in phase 5 (representing the post-ovulatory rise in progesterone). Overall, cycles in women with PMOS were less likely to be labeled ovulatory based on these thresholds, in line with the expectation that women with PMOS have fewer ovulatory cycles [22] (Fig 4F).

## Discussion

In this study, we demonstrated that arHMMs offer a novel biologically grounded approach for modeling the menstrual cycle using quantitative self-tracked urinary reproductive hormone data. HMMs provide a computational framework for modeling menstrual-hormonal physiology, allowing for variability in cycle length and in the timing of key physiological events across the cycle. The autoregressive component allows the model to leverage day-to-day trajectories within each cycle phase (e.g., rising or falling) rather than relying on absolute hormone values, which are variable both within and between women. Compared with a null model consisting of a Naive Bayes classifier, the arHMM framework demonstrated superior classification performance, illustrating the value of capturing hormone dynamics.

We further demonstrated the potential of arHMM-derived features to reveal distinct differences in hormonal trajectories between healthy controls and women with self-reported PMOS. Specifically, women with PMOS exhibited lower LH levels in phase 3 (periovulatory phase), and lower PDG levels in Phases 5 and 6 (mid-luteal and late luteal phases). While previous studies have observed higher LH levels during the follicular phase in women with PMOS relative to healthy controls (which we also observed, though non-significant), the lower average LH levels in Phase 3 that we observed in women with PMOS may reflect an underlying dysregulation in hormonal feedback as a result of poor follicle development and anovulatory hormonal patterns [23–25]. E_1_3G was also on average higher across the phases in women with self-reported PMOS, though these patterns were less clear, likely reflecting the heterogeneity of PMOS phenotypes present in this population [26,27]. The recent name change more accurately reflects the multisystem pathophysiology of PMOS, with broad clinical features including reproductive, metabolic, psychological and dermatological features [5]. The PMOS classifier performance was limited by the relatively small sample available for model development and the use of self-reported PMOS status. Further development and validation of classifiers based on arHMM-derived features will require larger datasets with clinically well-characterized and diagnostically confirmed PMOS phenotypes. Such studies will be important for determining whether these hormonal features can improve PMOS classification and achieve clinically meaningful predictive performance.

Finally, we explored several clinical applications of arHMM-derived features. We examined differences in hormone concentrations based on arHMM-derived phase information compared to cycle day. This approach has the potential to improve the timing of hormonal testing to permit a more precise determination of hormonal levels based on underlying hormonal trajectories. Using arHMM-derived phase information may permit clinicians to provide a more personalized assessment of underlying hormonal health, especially for individuals with atypical or long cycles [28]. We also examined the potential of arHMM-derived phase information to estimate a cycle’s ovulatory status based on PDG levels after the LH surge. With further research, this approach may offer a non-invasive means of determining whether an individual is experiencing anovulatory cycles. As ovulatory status is the most accurate reflection of underlying menstrual-hormonal health [29], there is a critical need for developing accessible and non-invasive methods for evaluating ovulatory status in an ongoing and dynamic way [7]. Further development of this approach also holds the potential of providing real-time feedback to individuals based on self-tracked hormonal data.

The arHMM also has important applications for menstrual cycle research. There is no universally accepted method for measuring the menstrual cycle as a continuous process [30]. Cycle-day-based approaches tend to oversimplify underlying hormonal dynamics and fail to capture individual variability in ovulation timing, which can lead to misidentification of important physiological events. Recent studies have shown the utility of dividing the menstrual cycle into sub-phases as a means of providing greater insight into the underlying hormonal environment [30,31]. The arHMM allows for identification of these latent sub-phases based on quantitative patterns and can be applied more broadly to map hormonal data to relevant menstrual-physiological states. Increasingly, menstrual phase is incorporated as an important predictor in biobehavioral research, creating a need for consistent and precise methods for determining menstrual phase, like the arHMM described here [2,17,32].

### Limitations

In this study, we demonstrated the potential of the arHMM framework for modeling menstrual cycle dynamics using self-tracked hormonal data. However, several limitations should be considered when interpreting the findings. This study was a secondary analysis of self-tracked urinary hormone data. The observational nature of these data introduces challenges related to missingness and uneven sampling. Because data collection occurred outside of a controlled research setting, participants were more likely to record hormone levels during the mid-cycle days, when hormonal monitoring is most relevant for predicting and confirming ovulation. As a result, data from the early follicular and late luteal phases were relatively sparse, limiting our ability to draw robust conclusions about hormonal patterns during these time points.

An additional limitation of this study is the limited availability of clinical and diagnostic information, particularly with respect to confirmation of a PMOS diagnosis. Inclusion in the PMOS group was based on participant self-report rather than established diagnostic criteria, introducing the potential for misclassification. Although self-report is commonly used in large-scale population-based research and may be the only feasible approach in real-world datasets [33], it lacks the diagnostic precision needed to distinguish PMOS from other conditions with overlapping menstrual cycle characteristics. Nevertheless, preliminary analyses demonstrated expected differences in cycle length between the self-reported PMOS and healthy control groups, providing some initial support for the validity of the group classifications. Moreover, the healthy control group exhibited hormonal trajectories that are consistent with established menstrual hormonal physiology indicating that the dataset offered a reasonable starting point for evaluating the feasibility of applying arHMMs to predict menstrual cycle phases. However, the results should be interpreted as an initial proof of concept rather than definitive validation of the approach. Future studies incorporating clinically confirmed diagnoses, standardized phenotyping, and more comprehensive clinical data will be essential to establish the generalizability and clinical validity of arHMM-derived menstrual cycle phase classifications.

## Conclusion

The integration of probabilistic modeling with home-based quantitative hormone tracking represents an important step toward scalable, data-driven menstrual health assessment. Home-based devices capable of quantifying urinary E_1_3G, LH and PDG metabolites enable frequent, high-resolution sampling that captures intra- and inter-cycle variability. When paired with the arHMM framework, these data provide individualized insight into hormonal dynamics, offering a window into reproductive physiology that is both accessible and physiologically meaningful. Such approaches may facilitate longitudinal tracking of hormonal health, helping women and clinicians identify changes in ovulatory function or signs of endocrine dysfunction over time. Although additional validation in larger and more diverse cohorts is needed, the findings highlight the potential of combining home-based hormonal testing with advanced time-series modeling to reduce diagnostic delays, improve access to PMOS screening, and support personalized reproductive health monitoring.

### Code availability

All code for model training and application, analysis, and generation of figures is available at https://github.com/lasugden/arHMM-cycle-tracking/

## Supporting information

S1 Appendix Table A. HMM transition matrix: Transition probabilities from row state to column state. The arHMM enforces movement from phase 1 through phase 6 in order. **Table B. HMM Emission Parameters:** For each phase and each hormone, we enforce a slope of 1 (in order to maintain a linear trajectory over multiple days) and learn the intercept and standard deviation of the linear regression model. As an example, we expect an average day-over-day rise in LH of about 17 mIU/ml while in phase 3. **Table C. Caption:** For each cycle, for each hormone and phase, we calculate the mean, minimum, and maximum for all available hormone measurements within that phase of the cycle (cycle-level features described in Methods). The table presents the averages and standard deviations of these cycle-level features separated into healthy control (HC) normal length cycles and PMOS normal length cycles (24–38 days). P-values are listed as not significant (ns), p < 0.05 (*), p < 0.01 (**), and p < 0.001 (***). **Table D. Caption:** For each cycle, for each hormone and phase, we calculate the mean, minimum, and maximum for all available hormone measurements within that phase of the cycle (cycle-level features described in Methods). The table presents the averages and standard deviations of these cycle-level features separated into healthy control (HC) abnormal length cycles and PMOS abnormal length cycles. P-values are listed as not significant (ns), p < 0.05 (*), p < 0.01 (**), and p < 0.001 (***). **Figure A. Average hormone patterns over phases, all cycles:** Averaging over all healthy control cycles, and all recordings per phase within each cycle, the arHMM reproduces expected patterns: a rise in E_1_3G in phase 2 (late follicular) and phase 3 (ovulation), a rise in LH in phase 3 and subsequent fall in phase 4 (early luteal), and a rise in PDG in phase 5 (mid-luteal). **Figure B. arHMM fit to cycle data depends on cycle length but not PMOS status**: We use the viterbi algorithm to calculate a cycle-based probability of the observed hormone levels and the most probable path through the phase states. We use this as a measure of model fit. Longer cycles have lower probabilities, as is expected because of how this probability is calculated. The scatterplot above (with x-jitter to better visualize the probabilities for different cycle lengths) along with best-fit lines for healthy controls and individuals with PMOS illustrates that the arHMM does not differ in its ability to fit the cycles of these two populations.
(DOCX)
