# Supplementary material for: Modeling menstrual cycle phases from urinary hormone trajectories using an autoregressive hidden Markov model
Source: PLOS Digit Health. 2026 Nov 5;5(11):e0001502. doi: 10.1371/journal.pdig.0001502 (PMC13739174; doi:10.1371/journal.pdig.0001502)
Supplement: Supplementary file 1 [file pdig.0001502.s001.docx]

**S1 Appendix**

**Table A**

|  | **Start/End** | **Phase 1** | **Phase 2** | **Phase 3** | **Phase 4** | **Phase 5** | **Phase 6** |
| --- | --- | --- | --- | --- | --- | --- | --- |
| **Start/End** | 0 | **1** | 0 | 0 | 0 | 0 | 0 |
| **Phase 1** | 0 | **0.833** | **0.167** | 0 | 0 | 0 | 0 |
| **Phase 2** | 0 | 0 | **0.888** | **0.112** | 0 | 0 | 0 |
| **Phase 3** | 0 | 0 | 0 | **0.667** | **0.333** | 0 | 0 |
| **Phase 4** | 0 | 0 | 0 | 0 | **0.8** | **0.2** | 0 |
| **Phase 5** | 0 | 0 | 0 | 0 | 0 | **0.8** | **0.2** |
| **Phase 6** | **0.167** | 0 | 0 | 0 | 0 | 0 | **0.833** |

**HMM transition matrix:** Transition probabilities from row state to column state. The arHMM enforces movement from phase 1 through phase 6 in order.

**Table B**

| **Phase** | **Hormone** | **Slope** | **Intercept** | **Std Dev** |
| --- | --- | --- | --- | --- |
| 1 | LH | 1 | -0.343 | 6.563 |
| 2 | LH | 1 | 0.193 | 5.085 |
| 3 | LH | 1 | 17.024 | 24.123 |
| 4 | LH | 1 | -9.341 | 11.151 |
| 5 | LH | 1 | -0.308 | 7.587 |
| 6 | LH | 1 | 0.458 | 7.867 |
| 1 | E3G | 1 | -0.551 | 64.526 |
| 2 | E3G | 1 | 12.912 | 76.119 |
| 3 | E3G | 1 | 43.739 | 125.849 |
| 4 | E3G | 1 | -35.132 | 98.471 |
| 5 | E3G | 1 | 0.033 | 88.106 |
| 6 | E3G | 1 | -11.458 | 84.215 |
| 1 | PDG | 1 | -0.506 | 4.646 |
| 2 | PDG | 1 | -0.015 | 3.987 |
| 3 | PDG | 1 | 0.787 | 4.451 |
| 4 | PDG | 1 | 1.658 | 6.296 |
| 5 | PDG | 1 | 0.529 | 7.649 |
| 6 | PDG | 1 | -1.388 | 7.454 |

**HMM Emission Parameters:** For each phase and each hormone, we enforce a slope of 1 (in order to maintain a linear trajectory over multiple days) and learn the intercept and standard deviation of the linear regression model. As an example, we expect an average day-over-day rise in LH of about 17 mIU/ml while in phase 3.

**Table C**

| Phase | E3G HC  (ng/ml) | E3G PMOS  (ng/ml) | p-value | LH HC  (mIU/ml) | LH PMOS  (mIU/ml) | p-value | PDG HC (μg/ml) | PDG PMOS  (μg/ml) | p-value |
| --- | --- | --- | --- | --- | --- | --- | --- | --- | --- |
| Phase 1 average mean (SD)  [avg min (SD) – avg max (SD)] | **112.8 (98.99)**  [102.3 (98.81)- 123.69 (103.78)] | **130.79 (95.84)**  [119.43 (95.76)- 144.01 (105.75)] | ns  ns  ns | **4.93 (6.27)**  [4.25 (6.14)- 5.85 (7.23) | **5.21 (4.69)**  [4.42 (3.71)- 6.24 (6.78)] | ns  ns  ns | **7.68 (6.24)**  [6.0 (5.62)- 9.54 (8.5)] | **9.08 (6.45)**  [7.13 (5.94)- 11.37 (9.07)] | *  ns  ns |
| Phase 2 average mean (SD)  [avg min (SD) – avg max (SD)] | **138.89 (65.73)**  [69.2 (42.35)- 243.73 (121.6) | **140.21 (79.61)**  [66.28 (55.9)- 255.74 (142.82)] | ns  ns  ns | **4.52 (9.84)**  [2.62 (9.86)- 7.84 (10.36)] | **4.41 (2.39)**  [2.13 (1.55)- 8.37 (4.91)] | ns  ns  ns | **4.61 (4.37)**  [2.81 (3.73)-  7.28 (5.95)] | **5.39 (4.39)**  [3.17 (3.53)- 8.46 (6.11)] | ***  ns  *** |
| Phase 3 average mean (SD)  [avg min (SD) – avg max (SD)] | **280.42 (154.71)**  [197.61 (148.53)- 366.26 (195.27) | **291.38 (156.89)**  [208.87 (151.34)- 378.6 (197.99)] | ns  ns  ns | **26.21 (18.36)**  [13.46 (11.59)- 42.82 (32.84)] | **22.84 (15.36)**  [13.41 (11.89)- 35.9 (29.96)] | ***  ns  *** | **7.03 (6.26)**  [5.7 (6.1)-  8.54 (7.29)] | **7.95 (6.3)**  [6.75 (6.17)- 9.22 (7.16)] | **  **  ns |
| Phase 4 average mean (SD)  [avg min (SD) – avg max (SD)] | **183.12 (110.91)**  [144.26 (90.75)- 223.75 (156.79)] | **197.31 (117.44)**  [154.2 (96.79)- 244.39 (169.08)] | *  *  * | **8.98 (11.06)**  [6.12 (10.24)- 12.47 (14.14)] | **9 (12.11)**  [6.32 (10.91)- 12.22 (14.81)] | ns  ns  ns | **8.29 (6.91)**  [7.19 (6.71)- 9.44 (7.77)] | **9.1 (7.16)**  [8.04 (6.98)- 10.23 (8.03)] | *  *  * |
| Phase 5 average mean (SD)  [avg min (SD) – avg max (SD)] | **154.06 (86.66)**  [119.08 (79.46)- 200.59 (125.12)] | **169.22 (102.86)**  [135.34 (98.3)- 211.01 (137.54)] | **  ***  ns | **4.63 (8.08)**  [3.59 (8.08)- 6.01 (8.58)] | **4.81 (8.73)**  [3.83 (8.4)- 6.07 (9.36)] | ns  ns  ns | **15.38 (7.41)**  [10.05 (8.09)- 21.34 (9.06)] | **15.36 (7.57)** [10.63 (8.39)-20.65 (9.04)] | ns  ns  ns |
| Phase 6 average mean (SD)  [avg min (SD) – avg max (SD)] | **134.86 (87.36)**  [107.09 (83.22)- 166.77 (107.59)] | **138.79 (89.51)**  [110.41 (85.23)- 171.16 (109.37)] | ns  ns  ns | **4.48 (8.52)**  [3.25 (7.83)- 6.48 (15.13)] | **4.73 (14.21)**  [3.7 (14.1)- 6.22 (14.88)] | ns  ns  ns | **14.58 (8.18)**  [10.66 (8.32)- 17.94 (9.34)] | **14.45 (8.45)**  [11.22 (8.73)- 17.21 (9.06)] | ns  ns  ns |

For each cycle, for each hormone and phase, we calculate the mean, minimum, and maximum for all available hormone measurements within that phase of the cycle (cycle-level features described in Methods). The table presents the averages and standard deviations of these cycle-level features separated into healthy control (HC) normal length cycles and PMOS normal length cycles (24-38 days). P-values are listed as not significant (ns), p<0.05 (*), p<0.01 (**), and p<0.001 (***).

**Table D**

| Phase | E3G HC  (ng/ml) | E3G PMOS  (ng/ml) | p-value | LH HC  (mIU/ml) | LH PMOS  (mIU/ml) | p-value | PDG HC (μg/ml) | PDG PMOS  (μg/ml) | p-value |
| --- | --- | --- | --- | --- | --- | --- | --- | --- | --- |
| Phase 1 average mean (SD)  [avg min (SD) – avg max (SD)] | **126.18 (111.61)**  [112.51 (113.61)- 140.31 (115.79)] | **122.59 (96.57)**  [113.83 (96.17)- 135.59 (108.44)] | ns  ns  ns | **5.91 (18.49)**  [4.92 (18.29)- 7.32 (19.36) | **4.67 (5.51)**  [3.83 (3.21)- 6.22 (13.9)] | ns  ns  ns | **7.89 (6.9)**  [5.72 (6.04)- 10.92 (10.02)] | **6.32 (5.81)**  [5.34 (5.33)- 7.83 (8.05)] | *  ns  *** |
| Phase 2 average mean (SD)  [avg min (SD) – avg max (SD)] | **140.29 (80.34)**  [68.71 (55.79)- 246.55 (131.97) | **133.53 (80.6)**  [62.19 (60.8)- 250.02 (136.5)] | ns  *  ns | **4.69 (7.71)**  [2.37 (3.18)- 8.31 (11)] | **5.14 (4)**  [2.39 (2.41)- 9.7 (7.03)] | ns  ns  ** | **5.12 (4.49)**  [3.05 (3.79)-  8 (5.94)] | **5.01 (4.13)**  [2.78 (3.32)- 8.35 (5.85)] | ns  ns  ns |
| Phase 3 average mean (SD)  [avg min (SD) – avg max (SD)] | **268.94 (148.78)**  [192.04 (142.93)- 348.17 (187.29) | **274.59 (161.87)**  [204.16 (154.11)- 348.62 (201.95)] | ns  ns  ns | **24.61 (17.49)**  [14.2 (13.34)- 39.3 (36.22)] | **21.87 (17.4)**  [12.84 (12.54)- 34.45 (32.59)] | **  *  ** | **7.53 (6.25)**  [6.07 (5.92)- 9.02 (7.37)] | **7.48 (6.18)**  [6.36 (6.03)- 8.8 (7.13)] | ns  ns  ns |
| Phase 4 average mean (SD)  [avg min (SD) – avg max (SD)] | **187.74 (121.44)**  [150.04 (107.38)- 226.26 (157.38)] | **193.67 (127.86)**  [156.18 (111.45)- 231.52 (165.39)] | ns  ns  ns | **10.19 (22.65)**  [7.45 (22.4)- 13.57 (24.45)] | **9.65 (15.03)**  [6.79 (14.21)- 13.15 (18.06)] | ns  ns  ns | **8.89 (7.27)**  [7.7 (7.17)- 10.18 (8.12)] | **8.62 (7.23)**  [7.53 (7.02)- 9.75 (8.07)] | ns  ns  ns |
| Phase 5 average mean (SD)  [avg min (SD) – avg max (SD)] | **171.79 (123.87)**  [136.01 (116.4)- 216.25 (152.25)] | **175.01 (121.31)**  [140.18 (115.64)- 221.83 (155.94)] | ns  ns  ns | **6.35 (23.59)**  [5.43 (23.63)- 7.58 (23.66)] | **5.91 (14.48)**  [4.77 (13.4)- 7.29 (14.99)] | ns  ns  ns | **15.68 (7.95)**  [10.56 (8.55)- 21.31 (9.36)] | **13.61 (8.07)** [9.38 (8.11)-18.62 (10.19)] | ***  **  *** |
| Phase 6 average mean (SD)  [avg min (SD) – avg max (SD)] | **150.69 (116.96)**  [118.64 (108.62)- 187.09 (135.69)] | **151.42 (107.99)**  [124.68 (102.98)- 182.95 (127.83)] | ns  ns  ns | **5.12 (15.33)**  [3.9 (14.8)- 7.16 (19.89)] | **5.56 (16.73)**  [4.59 (16.73)- 6.83 (17.02)] | ns  ns  ns | **14.62 (8.62)**  [11.16 (8.81)- 17.94 (9.49)] | **12.25 (8.4)**  [9.15 (8.1)- 15.35 (9.58)] | ***  ***  *** |

For each cycle, for each hormone and phase, we calculate the mean, minimum, and maximum for all available hormone measurements within that phase of the cycle (cycle-level features described in Methods). The table presents the averages and standard deviations of these cycle-level features separated into healthy control (HC) abnormal length cycles and PMOS abnormal length cycles. P-values are listed as not significant (ns), p<0.05 (*), p<0.01 (**), and p<0.001 (***).

**Figure A**

**Average hormone patterns over phases, all cycles:** Averaging over all healthy control cycles, and all recordings per phase within each cycle, the arHMM reproduces expected patterns: a rise in E_1_3G in phase 2 (late follicular) and phase 3 (ovulation), a rise in LH in phase 3 and subsequent fall in phase 4 (early luteal), and a rise in PDG in phase 5 (mid-luteal).

**Figure B**

**arHMM fit to cycle data depends on cycle length but not PMOS status**: We use the viterbi algorithm to calculate a cycle-based probability of the observed hormone levels and the most probable path through the phase states. We use this as a measure of model fit. Longer cycles have lower probabilities, as is expected because of how this probability is calculated. The scatterplot above (with x-jitter to better visualize the probabilities for different cycle lengths) along with best-fit lines for healthy controls and individuals with PMOS illustrates that the arHMM does not differ in its ability to fit the cycles of these two populations.
